# Neuroprotection from NMDA excitotoxic lesion by Cu/Zn superoxide dismutase gene delivery to the postnatal rat brain by a modular protein vector

**DOI:** 10.1186/1471-2202-7-35

**Published:** 2006-04-25

**Authors:** Hugo Peluffo, Laia Acarin, Anna Arís, Pau González, Antoni Villaverde, Bernardo Castellano, Berta González

**Affiliations:** 1 Unitat d'Histologia, Torre M5, Facultat de Medicina, Departament de Biologia Cel.lular, Fisiologia i Immunologia, and Institut de Neurociències, Universitat Autònoma de Barcelona, 08193, Spain; 2 Institut de Biotecnologia i de Biomedicina and Departament de Genètica i de Microbiologia, Universitat Autònoma de Barcelona, 08193, Spain

## Abstract

**Background:**

Superoxide mediated oxidative stress is a key neuropathologic mechanism in acute central nervous system injuries. We have analyzed the neuroprotective efficacy of the transient overexpression of antioxidant enzyme Cu/Zn Superoxide dismutase (SOD) after excitotoxic injury to the immature rat brain by using a recently constructed modular protein vector for non-viral gene delivery termed NLSCt. For this purpose, animals were injected with the NLSCt vector carrying the Cu/Zn SOD or the control GFP transgenes 2 hours after intracortical N-methyl-D-aspartate (NMDA) administration, and daily functional evaluation was performed. Moreover, 3 days after, lesion volume, neuronal degeneration and nitrotyrosine immunoreactivity were evaluated.

**Results:**

Overexpression of Cu/Zn SOD transgene after NMDA administration showed improved functional outcome and a reduced lesion volume at 3 days post lesion. In secondary degenerative areas, increased neuronal survival as well as decreased numbers of degenerating neurons and nitrotyrosine immunoreactivity was seen. Interestingly, injection of the NLSCt vector carrying the control GFP transgene also displayed a significant neuroprotective effect but less pronounced.

**Conclusion:**

When the appropriate levels of Cu/Zn SOD are expressed transiently after injury using the non-viral modular protein vector NLSCt a neuroprotective effect is seen. Thus recombinant modular protein vectors may be suitable for in vivo gene therapy, and Cu/Zn SOD should be considered as an interesting therapeutic transgene.

## Background

The pathobiology of acute damage to the CNS includes production of the superoxide anion (O_2_^-.^) and other reactive oxygen species that rapidly induce oxidative injury by lipid peroxidation, DNA damage and protein nitration [1] Many studies in the last decade have focused on the study of antioxidant proteins dealing with oxidative stress in physiological conditions and after injury.

Superoxide dismutases are among the most important cellular mechanisms that cope with oxidative stress. In normal conditions, cytosolic copper zinc superoxide dismutase (Cu/Zn SOD) and mitochondrial manganese superoxide dismutase (Mn SOD) are responsible for maintaining low levels of intracellular O_2_^-. ^by catalyzing its dismutation to oxygen and H_2_O_2 _[2]. In neuronal cells, endogenous Cu/Zn SOD is normally expressed but is rapidly downregulated after several types of acute brain insults [3-6] rendering the brain more susceptible to oxidative stress. In agreement with its antioxidant role, over-expression of Cu/Zn SOD in adult transgenic rats show pronounced neuroprotection in most acute CNS injury models [7-9] and targeted deletions of the Cu/Zn SOD gene or extracellular SOD genes worsens the outcome after focal ischemia in the adult brain [10,11].

After hypoxic/ischemic injury to the immature CNS, contradictory results have been reported in regards to the role of Cu/Zn SOD. Brain damage as a result of perinatal hypoxic-ischemic insult is a serious clinical problem with severe neurological consequences, where oxidative stress is known to play a fundamental role [12,13]. In previous studies using N-methyl-D-aspartate (NMDA)-mediated excitotoxicity, a model for hypoxic-ischemic injury to the postnatal brain [14,15], we have shown an upregulation of inducible nitric oxide synthase (iNOS) and cyclooxygenase-2 (COX-2) [16] and increased levels of the oxidative marker nitrotyrosine [17]. However, while a slightly worsened neuropathological outcome was observed in postnatal transgenic mice over-expressing Cu/Zn SOD [18,19], several antioxidant molecules including SOD mimetics like O_2_^-. ^dismuting metalloporphyrins have been shown to be neuroprotective [20]. In this sense, several differences between immature and adult animals in terms of oxidative stress and antioxidant defenses have been described including: upregulation of gluthathione peroxidase in the damaged adult brain but not in the damaged immature brain after trauma [21], the rapid free iron accumulation within 4 hours after transient cerebral ischemia stimulating Fenton reactions in the immature brain [22,23], the lesser concentration of metallothioneins, potent metal-binding antioxidant enzymes in the immature brain [24,25]. Finally, the postnatal brain is more sensitive than the adult brain to the neurotoxic actions of NMDA [26] which will lead to increased O_2_^-. ^generation [27,28]. In view of these contradictory findings of Cu/Zn SOD expression, we induced a transient post-injury overexpression of Cu/Zn SOD after excitotoxic damage to the immature rat brain using a novel non-viral gene therapy approach.

The design of non-viral vectors showing transgene expression in the brain has recently gained interest [29-32] due to the limitations imposed by viral vectors [33,34]. Non-viral modular approaches for gene therapy vectors based on the combination of several functional domains in a single polypeptide chain are of particular interest because of recombinant DNA methodologies that allow tailored designed vectors. The production of such protein vectors also permits a convenient scaling up and the vehicles exhibit high stability suitable for therapeutic uses. We have previously reported the construction of a recombinant modular protein vector that combines different functional domains displayed by E. coli β-galactosidase then engineered to conveniently accommodate a poly-lysine tail with DNA condensing/attaching properties and an integrin targeting RGD motif with cell attachment and internalization properties [35,36]. The resulting vector was capable of transferring a transgene to the intact and lesioned brain without any detectable acute inflammatory reaction or immune activation [29]. We have further improved the transfection efficiency of this modular vector by introducing the nuclear localization motif of the SV40 virus, generating the vector NLSCt [37].

The aim of this study was to assess the potential efficacy of the recombinant modular protein vector NLSCt complexed to the Cu/Zn SOD gene to improve neurological outcome after an acute excitotoxic injury to the immature brain.

## Results

### Lesion volume and neuronal cell death

The injection of NMDA into the sensorimotor cortex of the postnatal brain induces a well-characterized excitotoxic lesion [38] which includes the sensorimotor cortex, the dorso-medial striatum, and the rostral hippocampus.

**Figure 1:**
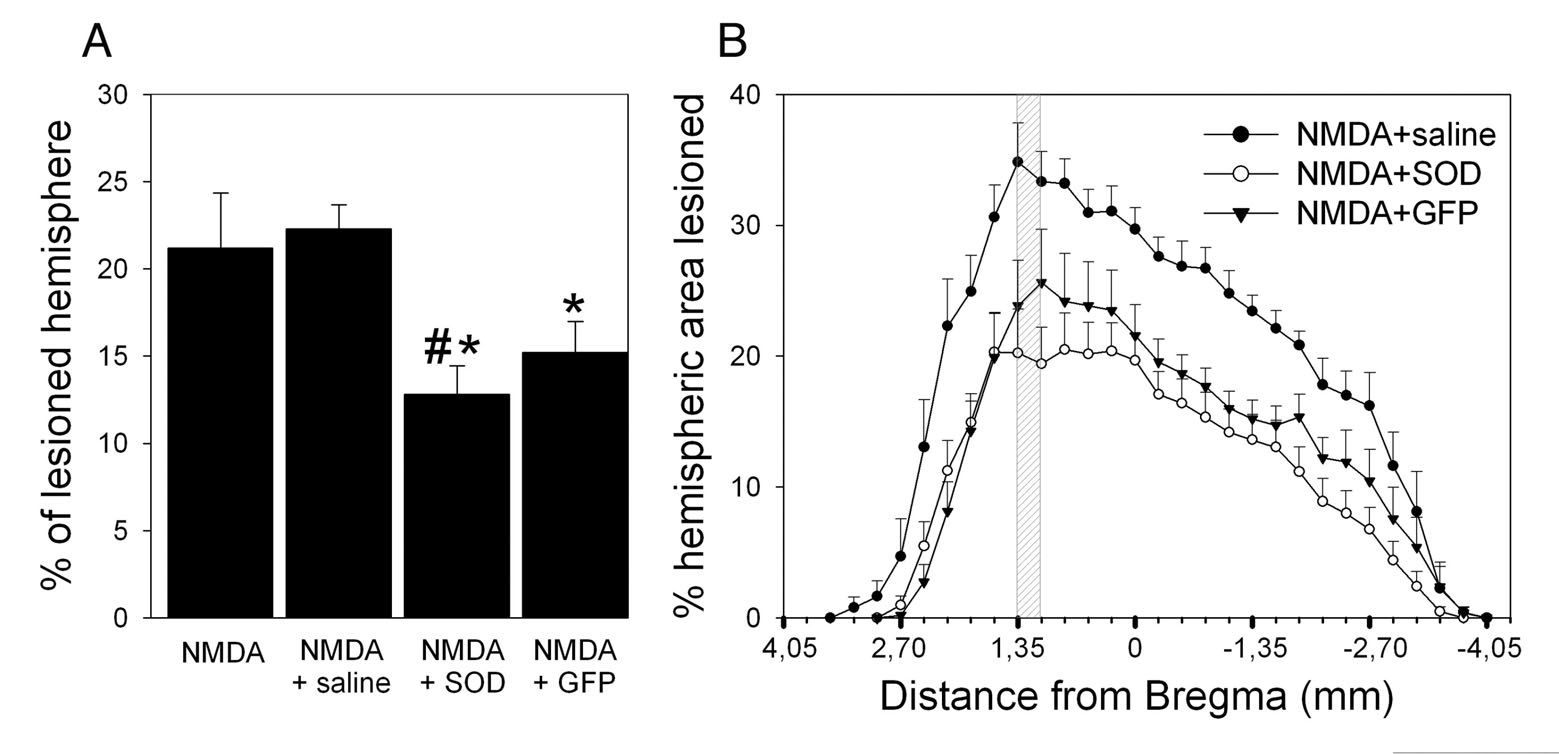
**Post-lesion over-expression of Cu/Zn SOD is neuroprotective**. NMDA lesioned animals were not re-injected or re-injected 2 hours after with either saline solution, the NLSCt vector carrying the transgene for Cu/Zn SOD or the NLSCt vector carrying the transgene for EGFP (NMDA, NMDA+saline, NMDA+SOD and NMDA+GFP respectively). The percentage of total lesioned hemisphere observed in A show that animals injected with the transgene for Cu/Zn SOD displayed a significant reduction in lesion volume in comparison with NMDA+saline (*p < 0.05) or in comparison to NMDA alone (#p < 0.05). The lesion volume in NMDA+saline injected animals was 36.2 mm^3^. Noticeably, animals injected with the GFP transgene also displayed a significant reduction, though only when compared to NMDA+saline injected animals; a reduction that was less pronounced than those found in Cu/Zn SOD injected animals. The rostro-caudal percent of lesioned hemispheric area is shown in B. The overall lesion was reduced at all levels of the brain of Cu/Zn SOD and GFP injected animals. The injection site is highlighted in grey.

**Figure 2:**
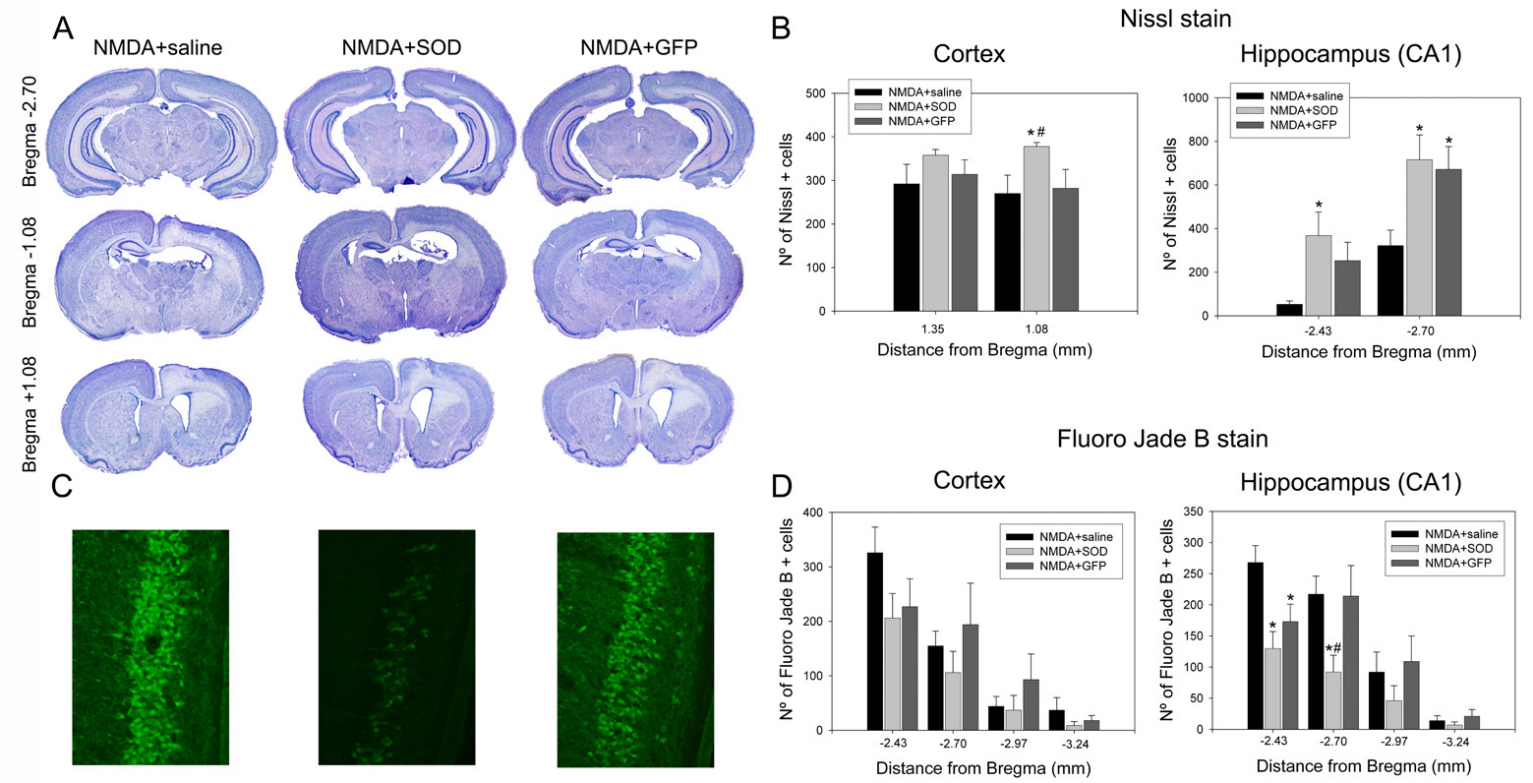
**Post-lesion over-expression of Cu/Zn SOD increases neuronal survival**. Nissl stained sections showing the lesion distribution in the animals post-injected with saline (NMDA+saline), NLSCt+Cu/Zn SOD (NMDA+SOD), or NLSCt+GFP (NMDA+GFP)(A). Quantification of Nissl stained neurons in the sensorimotor cortex and hippocampal CA1 penumbra showed increased cell survival in Cu/Zn SOD overexpressing animals (B). GFP overexpressing animals only showed an increase in neuronal number in hippocampal CA1 at a greater distance from the lesion point (B). Degenerating neurons were stained with Fluoro-Jade B staining (C), and quantitative analysis showed a significant reduction of degenerating neurons in Cu/Zn SOD overexpressing animals (D). (*p < 0.05 in relation to NMDA+saline injected animals and #p < 0.05 in relation to NMDA+GFP injected animals).

Rat brains injected two hours after the lesion with the NLSCt vector carrying the antioxidant enzyme Cu/Zn SOD transgene (NMDA+Cu/Zn SOD group) showed a pronounced reduction in the neurodegenerative area when compared to both the NMDA only group and the NMDA+saline group. The percentage of lesioned hemisphere volume was reduced by 42.6% in comparison with NMDA+saline animals (Figure 1A). The distribution of the lesion along the antero-posterior axis showed that the reduction in the neurodegenerative area extended rostrocaudally to all affected regions including cortex, striatum and hippocampus (Figure 1B, 2A). Both primary lesioned areas such as the sensorimotor cortex, and secondary lesioned areas like the caudal sub-plate neuronal layer, striatum and hippocampus showed a reduction in the extent of neurodegeneration. Surprisingly, the negative control, with the NLSCt vector carrying the GFP transgene (NMDA+GFP group), showed a less pronounced but significant reduction in the neurodegenerative area. The NMDA+GFP group was only significantly different from the NMDA+saline group and not the NMDA only group (the % of lesioned hemisphere volume was reduced by 29.4% with respect to the NMDA+saline group, Figure 1A, B, 2A).

To confirm the reduction in the extent of neurodegeneration, remaining neurons (by Nissl staining) and degenerating neurons (by FluoroJade B staining) present in various secondary degenerating regions were quantified. In NMDA+Cu/Zn SOD animals, the number of Nissl stained neurons in secondary degenerating regions such as, the lateral sensorimotor cortex where higher than those observed in NMDA+GFP animals. NMDA+Cu/Zn SOD animals also showed increased neuroprotection at the caudal region of hippocampal CA1 layer (Figure 2A, B). Accordingly, in NMDA+Cu/Zn SOD animals, Fluoro-Jade B stained degenerating neuron cell counts showed a significant reduction in the number of stained neurons at the caudal region of hippocampal CA1 layer whereas a not significant reduction was seen in the temporal cortex (caudal border of lesion) (Figure 2C, D). In contrast, in the NMDA+GFP group similar numbers of Nissl stained neurons in the sensorimotor cortex were seen when compared to NMDA+saline animals. Moreover, the number of Nissl-stained neurons at the CA1 layer of the hippocampus was only significantly higher at a greater distance from the lesion site in NMDA+GFP animals when compared to NMDA+saline animals, showing less pronounced neuroprotection than the NMDA+Cu/Zn SOD animals (Figure 2B). Although NMDA+GFP animals displayed reduced numbers of FluoroJade B degenerating neurons in the hippocampal CA1 region in relation to the NMDA+saline group, the reduction occurred only to a limited extent when compared to NMDA+Cu/Zn SOD animals (Figure 2C, D).

To determine if the reduction in the neurodegenerative area observed after the treatment with the NLSCt vector carrying the control GFP transgene was due to the DNA molecule or intrinsic to the NLSCt vector, we injected lesioned rats with the naked NLSCt vector. Interestingly, the NLSCt vector alone induced a significant 31.2% reduction of lesion volume (data not shown), reproducing the results obtained after the treatment with the NLSCt vector carrying the GFP transgene.

### Tyrosine nitration

**Figure 3:**
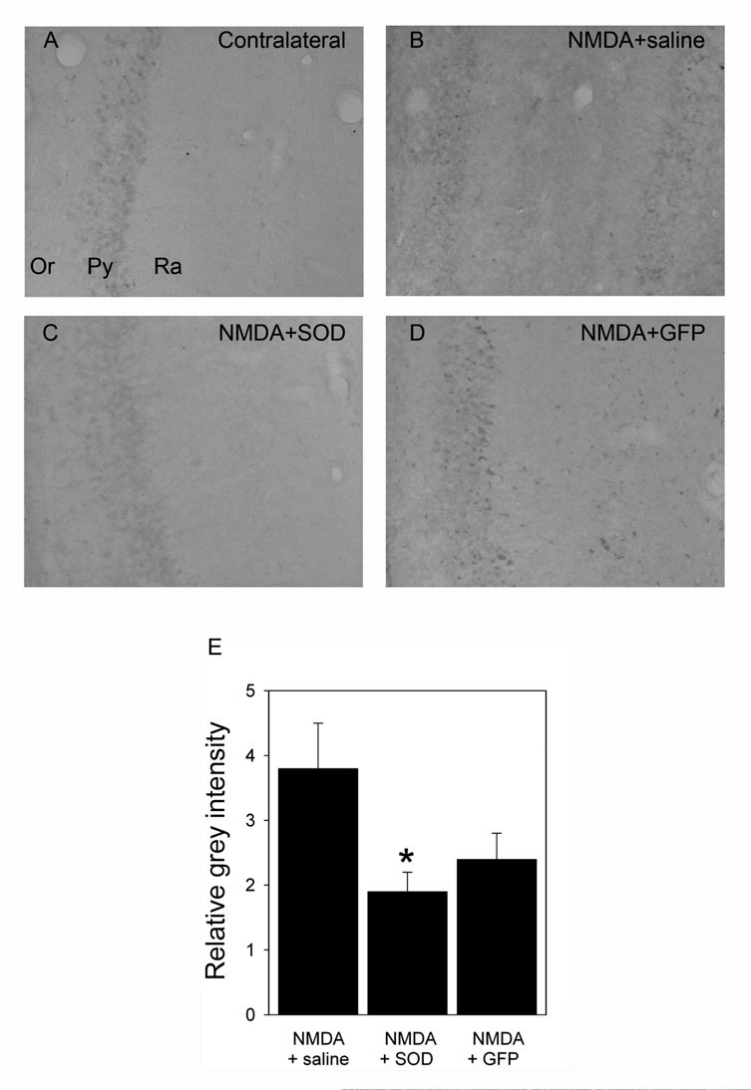
**Post-lesion overexpression of Cu/Zn SOD decreases nitrotyrosine immunoreactivity**. Sections from the caudal region of the CA1 hippocampal layer (bregma -2.43) of the contralateral side showed a basal nitrotyrosine immunoreactivity (A, Or: stratum oriens; Py; stratum pyramidalis; Ra: stratum radiatum). After NMDA injection, an increased immunorectivity was detected (B). However, animals overexpressing Cu/Zn SOD showed reduction in nitrotyrosine immunoreactivity (C), while animals overexpressing GFP showed a less pronounced reduction (D). Quantitative analysis confirmed these observations whereby, only animals overexpressing Cu/Zn SOD showed a significant (p < 0.05) decrease in nitrotyrosine immunoreactivity (E).

Protein tyrosine nitration is a footprint of peroxynitrite and other reactive species formation [39]. As we have previously described in detail [17], excitotoxic damage induces nitration in astrocytes and neuronal cells within the neurodegenerating area during the first 24 hours post-lesion and in the border of the lesion at longer survival times (Figure 3). In NMDA+Cu/Zn SOD animals, densitometrical measurements in a degenerating area at the border of the lesion such as the caudal region of the CA1 hippocampal layer showed a significant reduction in tyrosine nitration (immunoreactivity grade of 1.9 ± 0.3) in relation to NMDA+saline animals (immunoreactivity grade of 3.8 ± 0.7). Densitometry in the NMDA+GFP animals (2.4 ± 0.4) was not significantly different from the NMDA+saline group (Figure 3).

### Functional outcome

**Figure 4:**
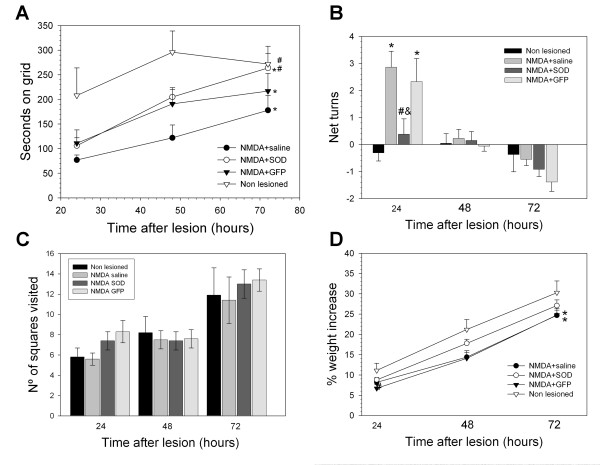
**Functional evaluation of NLSCt-Cu/Zn SOD injected animals**. At 24, 48 and 72 hours post-lesion, animals were subjected to several neurological tests including: the estimation of coordination skills by measuring the total climbing time until falling when placed on an inclinated grid (A); spontaneous turning behaviour in an open field (total turns recorded in 1.5 min.)(B); and spontaneous motor activity (C). Percentage of body weight increase was also followed (D). Lesioned animals injected with saline (NMDA+saline) showed a significant decrease in the time spent climbing on the inclinated grid in comparison to non-lesioned saline injected animals (Non lesioned)(A). Interestingly, only animals injected with the NLSCt vector carrying the Cu/Zn SOD transgene (NMDA+SOD) displayed a significant recovery in the time spent on the inclinated grid when compared to NMDA+saline animals (#p < 0.05). Animals injected with NMDA+saline or NMDA plus NLSCt vector carrying the EGFP transgene (NMDA+GFP) showed a significant increase in net turns compared to saline injected animals (*p < 0.05). In addition, only animals overexpressing Cu/Zn SOD showed a turning behaviour indistinguishable from non-lesioned control saline injected rats and significantly different from NMDA+saline (#p < 0.05) or NMDA+GFP injected animals (&p < 0,05)(B). There were no differences in the open field general motor activity of all experimental groups (C). Body weight of both NMDA+saline and NMDA+GFP injected animals showed a significant decrease in the developmentally physiological body weight increase in relation to saline injected animals (*p < 0.05). Overexpression of Cu/Zn SOD hindered this decrease and these animals showed an increase in body weight indistinguishable from control non-lesioned saline injected rats.

Interestingly, neurological tests carried out showed that only the Cu/Zn SOD overexpressing animals recovered significantly from the injury. In the inclinated grid climbing test, where general motor coordination is evaluated, only NMDA+Cu/Zn SOD animals showed significant and near complete recuperation of the time spent in the inclinated grid at 3 days, statistically indistinguishable from the non-lesioned saline injected animals (Figure 4A). Both NMDA+saline and NMDA+GFP injected animals showed a reduced performance in this task until 3 days, the last time analyzed. In addition to the improvement of the NMDA+SOD injected animals observed in the grid climbing test, the spontaneous turning behaviour, a neurological sign of un-balanced striatal neurotransmission, also showed a similar profile (Figure 4B). NMDA+saline and NMDA+GFP animals showed a significant spontaneous net turning toward the ipsilateral side at 1 day when compared with non-lesioned saline injected animals. In contrast, NMDA+Cu/Zn SOD animals did not show any bias on their net turning behaviour, not differing significantly from the non-lesioned saline injected rats. All groups performed equally well on an open field motor task (Figure 4C), demonstrating similar motor activity, which could not account for the differences observed in both of the neurological tests performed. In addition, a general improvement of the Cu/Zn SOD overexpressing rats could also be observed in the development-mediated daily weight increase (Figure 4D) where again, only the Cu/Zn SOD over-expressing animals were statistically indistinguishable from the non-lesioned saline injected controls.

## Discussion

This study shows for the first time that consistent functional and neuropathological recovery from acute immature brain damage can be achieved by post-lesion overexpression of the Cu/Zn SOD antioxidant enzyme delivered through a modular multifunctional protein vector. This treatment protected against excitotoxic damage, thought to be an important mechanism underlying neuronal death after hypoxic/ischemic injury to the neonate [13,40], but also in acute adult brain neurodegenerative conditions such as stroke [41] and traumatic brain injury [42], as well as in chronic ones as Alzheimer's [43], Parkinson's [44] and Huntington's [45] disease.

### Cu/Zn SOD as a therapeutic transgene

In this study, the Cu/Zn SOD transgene was expressed under the control of the cytomegalovirus immediate early promoter and as such, expressed in any cell type. We have previously shown that after postnatal excitotoxic injury, neurons, astrocytes and microglial cells are the main cell types showing transgene expression mediated by NLSCt-type vectors [29]. Under physiological conditions, Cu/Zn SOD is mainly expressed in neurons [6,46], however, in our experimental conditions several glial cell types are also transfected [29] and could indirectly mediate the neuroprotective effect. Noteworthy, O_2_^-. ^produced after damage in both neurons and glial cells can be dismutated to H_2_O_2 _by Cu/Zn SOD activity, hindering the formation of the potent oxidant and protein nitrating agent peroxynitrite [47,48]. The reduction in the levels of nitrotyrosine reported here support this mechanism of Cu/Zn SOD neuroprotection. Interestingly, there is a great deal of evidence that suggests that O_2_^-.^/peroxinitrite species toxicity is higher than that of H_2_O_2_. A study of Cu/Zn SOD overexpressing astrocytes exposed to O_2_^-. ^found that they had higher survival rates than control astrocytes even when glutathione peroxidase and catalase activities were blocked and GSH levels depleted [49]. Cu/Zn SOD overexpressing astrocytes also survived better that control astrocytes after oxygen glucose deprivation, in the absence of glutathione peroxidase upregulation and with a lower catalase upregulation in comparison to control astrocytes [50]. These Cu/Zn SOD overexpressing astrocytes, unlike controls, also maintained elevated GSH concentration. Peroxinitrite but not H_2_O_2 _has also been shown to trigger an in vitro reactive phenotype of astrocytes that is toxic for co-cultured motor neurons [51]. These data suggest that overproduction of H_2_O_2 _is not a major factor in astrocytic injury. On the other hand, specific scavenging of O_2__-. _can also increase neuronal survival under some pathologically relevant conditions. For example, motor neurons can be rescued from trophic factor withdrawal by liposome-mediated Cu/Zn SOD protein delivery [52] and synthetic SOD mimetics [53,54]. Neuronal cultures can also be protected from excitotoxicity by SOD mimetics [55], adenovirally mediated overexpression of Cu/Zn SOD [56], or transgenically overexpressed Cu/Zn SOD [57,58]. However, in some particular cases, Cu/Zn SOD overexpression can reduce neuronal survival during direct extracellular exposure to superoxide generators by a mechanism involving excess H_2_O_2 _accumulation [59], while protecting from direct H_2_O_2 _treatment [58]. Finally, some research suggests that O_2__-. _is more toxic than H_2_O_2 _by reacting with NO to form peroxinitrite [47,48] as been shown by inhibition of NO production in neuronal cultures submitted to an excitotoxic damage. This treatment is sufficient for inducing neuroprotection [60-62], while O_2__-. _and H_2_O_2 _are still being formed but will not be so toxic. Thus, the increase in Cu/Zn SOD expression in neurons and astrocytes most likely contributes to the neuroprotection observed in vivo.

Regarding lesion volume, we show a significant decrease in all NLSCt treated animals when compared to NMDA+saline injected animals, but no difference between these NLSCt treated groups. However, only the Cu/Zn SOD treated animals showed a significant reduction in lesion volume compared to NMDA alone lesioned animals, which is in fact the real control of the therapeutic gene therapy approach. It is important to consider the functional recovery induced by Cu/Zn SOD therapy in both of the neurological evaluation tests that could reflect increased preservation of functional synaptic contacts and white matter tracts. The reduction in the developmental increase in daily body weight observed in lesioned animals and GFP treated animals was not observed in Cu/Zn SOD treated animals, which also supports the general improvement of these animals.

In accordance with our findings, SOD mimetics (such as O_2__-. _dismuting metalloporphyrins) were shown to be neuroprotective after ischemia in the immature brain [20]. However, our results contrast with the previously reported exacerbation of hypoxic/ischemic injury occurring in immature transgenic mice over expressing Cu/Zn SOD [18]. Though the reason for this difference is not clear, several reasons besides species specificity and lesion model could contribute to its explanation. In our experimental conditions, the NLSCt vector induced a transient and lower level of Cu/Zn SOD transgene expression compared to the higher and permanent expression found in transgenic mice. It has previously been shown that very high levels of Cu/Zn SOD, as those observed in transgenic animals, can produce alterations such as, an increase in basal lipid peroxidation [63], mitochondrial vaquolation [64,65], abnormalities in neuromuscular junctions [66], or deficits in long-term potentiation (LTP) and spatial memory [67]. Furthermore, after life-long overexpression of Cu/Zn SOD, compensatory changes in the basal levels or induction of other antioxidant enzymes like Mn SOD [63], heme oxygenase [68], or glutathione peroxidase [69] have been documented that provide an altered redox balance in transgenic animals.

In regards to expression levels, it has been reported that polyethylene glycol-conjugated Cu/Zn SOD treatment after focal ischemia showed a U-shaped dose-response curve, implying that the effective neuroprotective dose of this enzyme may be in fact concentration restricted [70]. Therefore, several parameters like protein levels, time-course and cell population of Cu/Zn SOD expression could affect the overall outcome after the lesion and underlie the differences between gene therapy and transgenic mice approaches.

### NLSCt vector for neuroprotective gene therapy

Studies showing phenotypic or functional effects derived from transgene expression in the CNS are generally absent [71]. Regarding non-viral vectors, only one flexible liposome/antibody-conjugated non-viral vector has been reported to induce functional recovery, reversing motor abnormalities after a 6-hydroxydopamine striatal lesion [72].

Several multifunctional protein vectors have been developed by combining functional modules from different origins, driving the four main steps for successful DNA transfer to the cell nucleus: DNA ligation-condensation, cell attachment-internalization, endosome disruption-escaping, and nuclear import. Although many of these prototypes can transfect cells in culture, their efficiency in vivo is very limited (reviewed in [73]). We have previously shown that an earlier version of the NLSCt modular vector had a restricted capacity for transgene delivery to the intact brain. However, it was very efficient in conducting widespread transgene expression after an excitotoxic lesion [29], probably due to the disruption of the extracellular matrix and the small size of the vector/DNA complexes (20–40 nm diameter) [36]. In this study, we show that NLSCt had a very high transfection efficiency, only 24 ng of NLSCt-coated Cu/Zn SOD plasmid was able to reduce oxidative stress and rescue neurons from cell death in different areas of the lesion border, that considerably reduced the total lesion volume and increased functional outcome. This transfection efficiency could be due to integrin mediated endocytosis and enhanced transit towards the nuclear compartment mediated by the SV40 viral nuclear localization sequence of NLSCt [37]. It could also be due to massive neuronal endocytosis that reaches the nuclear compartment, a process previously described in neuronal cells within a few hours after injection of toxic and sub-toxic doses of NMDA or kainate [74,75].

Transient Cu/Zn SOD expression induced by NLSCt-mediated gene therapy seems to be an important parameter in conferring neuroprotection. In the same experimental model as the one used here, and also using the transgene regulated by the CMV promoter, the expression of the GFP transgene was transient, almost disappearing at 7 days postinjury [29]. Whereas a prolonged stable delivery of a transgene would be desirable for long-term improvement of pathologies such as inherited monogenic defects, transient transgene delivery may be more beneficial than a constitutive expression for therapeutic intervention after acute CNS damage.

Interestingly, both the NLSCt vector carrying a control transgene and the nude vector showed a significant grade of neuroprotection, indicating that the effect was intrinsic to the NLSCt vector. One of the bioactive motifs of NLSCt is the foot-and-mouth disease virus integrin-interacting RGD peptide, which interacts preferentially with different α_V _integrins [76]. This may suggest that our results with the vector show a neuroprotective effect mediated by the interaction between the RGD motif of NLSCt and specific integrins through an unknown mechanism. This is supported by a recent study showing that blocking αD/β2 integrins is strongly neuroprotective after spinal cord injury [77]. In addition, although the NLSCt vector inhibits the interaction of RGD dependent integrins with their natural extracellular matrix ligands, it could be directly activating integrin outside-in signaling events [78].

## Conclusion

We show that transient overexpression of Cu/Zn SOD after an excitotoxic injury to the immature rat brain using a modular protein for non-viral gene delivery can be neuroprotective and improve functional outcome; signalling this vector as a promising tool for in vivo gene therapy strategies for acute CNS lesions.

## Methods

### Protein, DNA and protein-DNA complexes

Protein NLSCt is an engineered form of Escherichia coli beta-galactosidase that displays an integrin-targeted RGD motif. This segment, inserted between residues 249 and 250 of the bacterial enzyme, reproduces the cell-attachment region of the VP1 capsid protein of foot-and-mouth disease virus [36] that binds host cells preferentially by integrin α_V_β3 but also by integrins α_V_β6, α_V_β5, and α_V_β8 [76]. The additional presence of a deca-lysine tail joined to the amino terminus of the construct and a still unidentified enzyme segment with nuclear targeting properties and the SV40 NLS at carboxi terminus of the recombinant protein [79] enables NLSCt to promote efficient DNA delivery. The NLSCt protein was produced in bacteria and purified from crude cell extracts as described previously [35]. The human Cu/Zn SOD gene cloned into the plasmid pcDNA3.1 (Invitrogen, Carlsbad, CA, USA) under the control of a cytomegalovirus immediate early promoter was used (generously provided by AG Estévez and JS Beckman). A red-shift variant of jellyfish Aequorea Victoria green fluorescent protein (GFP) gene encoded into plasmid pEGFP-C1 (Clontech, Palo Alto, CA, USA) under the control of the same cytomegalovirus promoter was used as a control. Protein and DNA complexes were formed by incubation in 0.9 % NaCl at room temperature for 1 hour at ratios of 0.03 μg DNA per μg of NLSCt protein. Details of complex formation are provided elsewhere [35].

### Excitotoxic injury and treatment paradigm

Nine-day old Long-Evans black-hooded rat pups (15–20 gr., both sexes; Janvier, France) were used. All intracerebral injections were made into the right sensorimotor cortex at the level of the coronal suture (2 mm lateral of bregma and 0.5 mm depth) using a stereotaxic frame adapted for new-borns (Kopf Instruments) under isoflurane (Baxter International Inc.) anesthesia. Excitotoxic lesions were performed as previously described [80], by injecting 18.5 nmol of N-methyl-D-aspartate (NMDA) (Sigma-Aldrich, St. Louis, MO, USA) diluted in 0.15 μl of saline solution (0.9 % NaCl) at a rate of 0.05 μl/min using an automatic injector. One microliter of either the NLSCt vector (0.8 μg/μl) carrying the control EGFP plasmid or the Cu/Zn SOD plasmid, the NLSCt vector alone (0.8 μg/μl), or the vehicle (NaCl 0.9 %) was injected 2 hours after the excitotoxic lesion at the same coordinates at 0.2 μl/min. After suture, pups were placed on a thermal pad for 2 hours at 36°C to maintain normothermia. Experimental animal work was conducted according to Spanish regulations in agreement with European Union directives. Experimental procedures were approved by the ethical commission of the Autonomous University of Barcelona. All efforts were made to minimize animal suffering.

### Behavioural and neurological testing

Quantitative methods for the evaluation of adult motor performance [81] have been adapted here for postnatal pups (P9–P12). These methods of scoring gave consistent values for different observers. From day 1 until day 3 post-lesion, all rats were weighed and tested once a day in each of the following tasks: general motor activity, net turns and inclinated grid climbing. For the evaluation of general motor activity and net turns, rats were placed in an open field (70 cm × 70 cm) immediately after the separation from the mother and their spontaneous activity was recorded as number of new squares (10 × 10 cm) visited. Simultaneously, the total number of spontaneous complete turns (ipsilateral = positive and contralateral = negative) were recorded. As previously shown elsewhere, un-lesioned animals as well as saline injected animals did not show turning bias towards either side. In this test, lesioned rats show spontaneous turning bias towards the ipsilateral side while the lesion over-stimulates the ipsilateral parenchyma. After one day it disappears due to the complete destruction of neurons and then turning can only be observed by injection of amphetamine. The inclinated grid climbing test was performed by allowing the rats to climb an inclined grid (metal bars of 3.5 mm in diameter and separated by 7.5 mm) at an angle of 45°. Climbing the grid is a spontaneous response. However, in the few cases when a rat stayed still on the grid, it was removed and placed again on the grid. The total time climbing on the grid until five consecutive falls occurred was recorded each day for every rat. The task was interrupted after 5 falls or after 360 seconds on the grid. Unlesioned animals and saline injected animals consistently climbed for longer time periods than lesioned animals.

### Histology and lesion volume measurement

Three days post-lesion, rats were anaesthetized and perfused intracardially with 4% paraformaldehyde in 0.1 M phosphate buffer (pH 7.4). Brains were post-fixed in the same fixative for 2 hours and sunk in a 30% sucrose solution before being frozen with dry CO_2_. Coronal sections of the entire brain (30-μm thick) were obtained using a Leitz cryostat. Parallel sections of the entire brain every 240 μm were collected directly on a slide, stained for Nissl and used for the measurement of the total lesioned area and total hemisphere area after high resolution digitizing. Using the analySIS^® ^software, the pale Nissl stained lesioned area was quantified by parallel observation of the slides on a microscope. To avoid misinterpretations due to possible tissue edema or postmortem alterations during cutting or mounting, data are presented as percentage of the ipsilateral hemisphere.

### Immunohistochemistry for nitrotyrosine and densitometrical analysis

Nitrotyrosine labeling, a marker of peroxynitrite and other reactive oxygen species formation, was used to evaluate the level of oxidative stress. Sections were processed for endogenous peroxidase inactivation and blocked for 1 hour in Tris-buffered saline (TBS, pH 7.4), 10 % fetal calf serum plus 1% Triton X-100. Sections were incubated overnight at 4°C in the same blocking solution with a primary antibody against nitrotyrosine (1:60)(06–284, Upstate Biotechnology, Lake Placid, NY, USA). After several washes they were incubated for 1 hour with biotinylated anti-rabbit (1:200, Amersham RPN-1004). Specific labeling was evidenced by incubation with avidin-peroxidase (1:400 Dakopatts P0364) for 1 hour and subsequent 3,3'-diaminobenzidine (DAB)-hydrogen peroxide developing procedure. For densitometrical measurements, digitalized images from ipsilateral lesion border (penumbra) and contralateral hippocampal CA1 regions were analyzed with the analySIS^® ^software for total grey intensity. Data was expressed as the ratio between immunolabeling intensity in the injured and contralateral hemispheres.

### Fluoro-Jade B staining

Neuronal degeneration was detected as previously described [82]. Briefly, free-floating sections were mounted and air dried overnight. After dehydration in ethanol (30%, 50%, 70%, 96% and 100%) and rehydration (etanol 96% and 70%), sections were rinsed with distilled water and oxidized with MnO4K (0.06% in water, 15 min.). Then sections were rinsed with distilled water, incubated with Fluoro Jade B (Histo-Chem, Inc. Jefferson, USA) (0.0004% in water plus 1% acetic acid glacial, 20 min.), washed with distilled water, air dried and mounted.

### Statistical analysis

All results are expressed as mean ± standard error mean (SEM). Six rat litters containing at least 3 different experimental groups were used. A total of 8 saline injected animals and 51 NMDA injected animals were used (NMDA only, n = 4; NMDA+saline, n = 14; NMDA+NLSCt/pSOD, n = 13; NMDA+NLSCt/pEGFP, n = 16; and NMDA+NLSCt, n = 4). ANOVA followed by Fisher's PLSD post-hoc test was used to determine significant differences (p < 0.05) in lesion volume, cell counts, nitrotyrosine densitometry and number of squares visited measurements. Repeated measures ANOVA followed by Fisher's PLSD post-hoc test were used to evaluate significant differences (p < 0.05) between groups in weight increase and in inclinated grid walking. Analysis of significant differences between groups in the measurements of the number of net turns was performed by ANOVA followed by Fisher's PLSD post-hoc test after ranking of the data.

## Authors' contributions

HP carried out the brain lesions and animal work, performed the immunohistochemical staining, the Fluoro Jade B staining and the behavioural studies, and also conceived the study and drafted the manuscript. LA participated in the design of the study and helped to draft the manuscript. PG helped with the behavioural studies, brain lesions and DNA production. AA produced and purified the vector and the DNA. AV helped to draft the manuscript. BC and BG coordinated and supervised the development of the study, were responsible for the project giving economical support and helped in the last version of the manuscript. All authors read and approved the final manuscript.
